# Cost effectiveness of the Texas wildlife rabies Border Maintenance Zone

**DOI:** 10.1371/journal.pntd.0014168

**Published:** 2026-04-01

**Authors:** Stephanie Shwiff, Glenn Swanson, Steven Shwiff, Mike Bodenchuk, Joanne Maki

**Affiliations:** 1 United States Department of Agriculture-Animal Plant Health Inspection Service-Wildlife Services, National Wildlife Research Center, Fort Collins, Colorado, United States of America; 2 Department of Management and Economics, Texas Agriculture &Mechanical University, Commerce, Texas, United States of America; 3 Texas Wildlife Services, San Antonio, Texas, United States of America; 4 Veterinary Public Health, Boehringer-Ingelheim Animal Health, Athens, Georgia, United States of America; University of Liverpool, UNITED KINGDOM OF GREAT BRITAIN AND NORTHERN IRELAND

## Abstract

Rabies prevention in the United States (US) relies on a robust public health infrastructure that includes pet vaccination, post-exposure prophylaxis (PEP), and wildlife surveillance, costing $245–$510 million annually but saving over $1 billion in avoided healthcare expenditures. While canine rabies has been eliminated from the US, wildlife reservoirs continue to pose a zoonotic threat, particularly bats, skunks, raccoons, and foxes. Oral rabies vaccination (ORV) programs targeting coyotes, foxes, and raccoons have demonstrated significant public health and economic benefits across multiple states, with benefit-cost ratios consistently above 1.0. In 2023, the Texas Oral Rabies Vaccination Program (TX-ORVP) reduced bait density within its ORV program’s barrier maintenance zone (BMZ) to lower fixed costs. This study applies cost-effectiveness analysis (CEA) to assess whether these reductions remain economically viable under scenarios involving increased variable costs. Three scenarios were modeled: (1) enhanced surveillance without contingency actions, (2) rural incursions requiring contingency actions, and (3) comprehensive breaches involving surveillance, contingency actions, and increased PEP. Results indicate that surveillance costs could increase by up to 155% before negating savings, but any scenario requiring contingency actions renders the BMZ2023 cost-inefficient compared to pre-2023 practices. These findings underscore that while reduced bait density may appear cost-saving, heightened risk of rabies incursions can quickly erode economic sustainability. Sustained investment in wildlife rabies ORV programs is necessary for effective and efficient long-term rabies control and public health protection.

## Author summary

Rabies remains a persistent zoonotic threat in the United States, despite the elimination of canine rabies through widespread dog vaccination programs. Today, over 90% of rabies cases occur in wildlife reservoirs such as bats, skunks, raccoons, and foxes, making eradication impossible. Public health infrastructure, including pet vaccination, post-exposure prophylaxis (PEP), and wildlife surveillance, has successfully limited human rabies deaths to fewer than ten annually, but at a cost exceeding $500 million per year. These investments save more than $1 billion annually by preventing unnecessary medical interventions. Wildlife rabies prevention programs, particularly oral rabies vaccination (ORV), have proven cost-effective in reducing spillover into domestic animals and humans. This study evaluates the economic sustainability of the 2023 Texas Oral Rabies Vaccination Program (TX-ORVP) barrier maintenance zone (BMZ), which reduced bait density to lower fixed costs. Using cost-effectiveness analysis (CEA), we examine whether potential increases in variable costs, such as viral surveillance, contingency actions, and PEP, could outweigh the savings. Our findings suggest that while the BMZ2023 may remain cost-effective under limited scenarios, any breach requiring contingency actions renders the program economically inefficient. This analysis highlights the importance of balancing short-term cost savings with long-term public health protection.

## Introduction

In the United States (US), canine rabies is no longer a public health threat due to state regulated dog vaccination programs. From 2012-2022, > 90% of US rabies cases were in wildlife with the majority occurring in four reservoir species (i.e., bats, skunks, raccoons, and foxes) [1–11]. Due to the presence of multiple host species in the US eradication of rabies is not possible [12]. Rabies prevention in the US is estimated by the Centers for Disease Control (CDC) to cost between $245 to $510 million/yr. [13]. Such efforts include vaccinating pets and administering post-exposure prophylaxis (PEP) to humans exposed to the rabies virus (RV). Each year hundreds of thousands of animals are observed or tested for rabies in the US and approximately 60,000 people require PEP. The CDC contends that with a solid public health infrastructure in place, human rabies deaths in the US have recently been kept to <10 per year. However, maintaining such rabies prevention systems costs more than $500 million/yr. Rabies prevention efforts in the US save over $1 billion in healthcare cost annually by accurately assessing exposure risk and thus preventing unnecessary medical costs [14]. While human rabies cases are rare in the US, recent human fatalities due to bat rabies underscore the on-going zoonotic public health risk of this disease [15]. Wildlife rabies prevention efforts lessen spillover of wildlife RVs into domestic species thus minimizing human exposure to this deadly virus.

**Fig 1:**
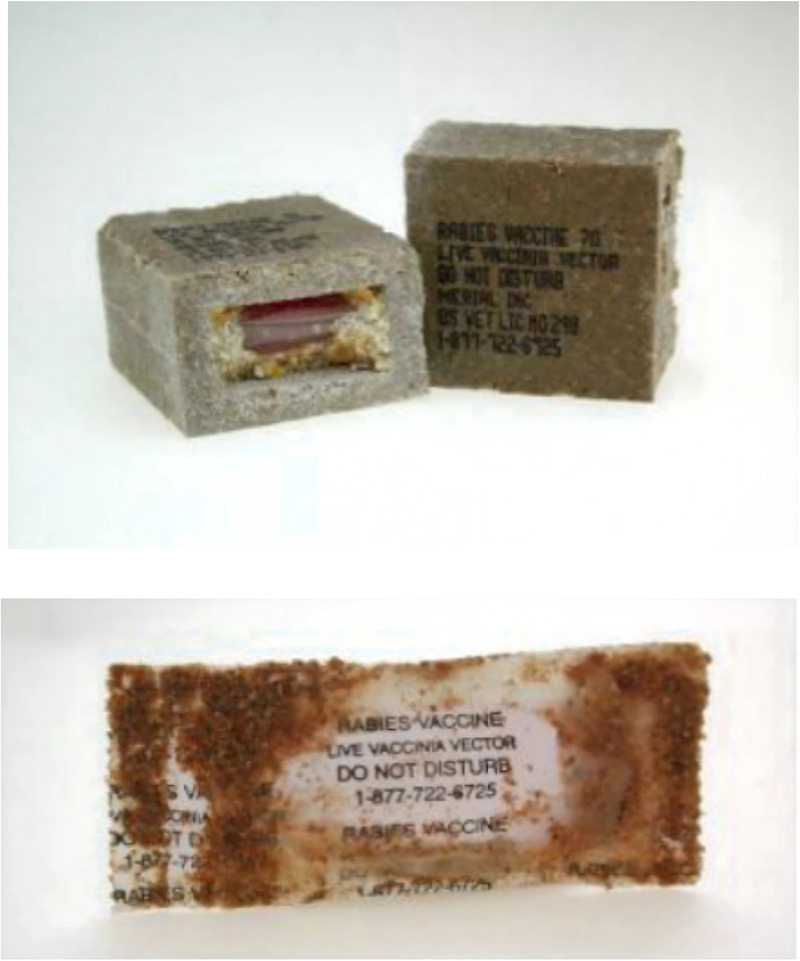
Photos of the RABORAL V-RG oral rabies vaccine product. The Texas Oral Rabies Vaccination Program has used RABORAL V-RG to vaccinate wildlife since 1995 [19]. From 1995 through 2013, the fishmeal polymer block bait (top) was distributed for coyotes and a chicken-based dog food block bait was distributed for gray foxes. Beginning in 2014, the coated sachet format with fishmeal coating (bottom) became the choice for both coyotes and foxes. The liquid vaccine dose is contained within a heat-sealed plastic sachet with labeling including a toll-free number for reporting purposes.

Oral rabies vaccines (ORV) were first used more than 30 years ago to eliminate red fox *(Vulpes vulpes)* rabies from Western Europe [16,17]. In the US, the same technology has proven to effectively vaccinate raccoons *(Procyon loto*r), coyotes *(Canis latrans)*, and gray foxes *(Urocyon cinereoargenteus)* against rabies. The ORV used in Texas since 1995 is a recombinant poxvirus-vectored virus expressing the rabies glycoprotein called RABORAL V-RG (Boehringer Ingelheim Animal Health, Inc., in Athens, Georgia, USA) [18]. Today, it is the only licensed wildlife rabies vaccine in the US. Each dose consists of a vaccine-filled sachet inserted into an edible fishmeal polymer block bait or coated with a fishmeal attractant (Fig 1) [20].

Due to the remoteness and rugged terrain of west Texas, ORV doses are distributed into wildlife habitat using low-flying airplanes and helicopters. Raccoons, foxes, and coyotes consuming the baits are vaccinated and within 4–6 weeks develop a protective rabies antibody response. Vaccinating wildlife within a geographically defined area creates an immune population which impedes RV transmission. Annual bait distribution campaigns revaccinate adult animals as well as vaccinate immunologically naïve juveniles.

The National Rabies Management Plan (NRMP), managed by the United States Department of Agriculture – Animal Plant and Health Inspection Service, Wildlife Services (USDA-APHIS-WS), partners with public health agencies, departments of natural resources, and the CDC. Primary NRMP activities include distributing ORV doses (4–5 million/yr), collecting sera from wildlife for detection of rabies antibodies, and monitoring vaccination zones for rabies outbreaks and translocated infected animals (i.e., enhanced surveillance). Longstanding ORV programs (ORVP) initiated in the mid-1990’s have prevented the western spread of the raccoon RV (R-RV) beyond the Appalachian Mountains and eliminated the domestic dog RV circulating in coyotes (DDC-RV) from the US [21]. The DDC-RV was eliminated from the US in 2007, and the last case of the Texas fox variant (TF-RV) was detected in a rabid cow in 2013 [3,22].

Due to the unpredictable nature of rabies outbreaks, NRMP agencies must be ready to respond quickly to wildlife rabies outbreaks anywhere in the US. For example, in 2024, USDA-WS and Nebraska public health offices responded to a translocated rabid kitten case in Omaha, Nebraska. A state-wide contingency action (CA) was implemented when the kitten’s RV was determined to be of raccoon origin [23]. The raccoon RV is not found in the central US where skunk rabies is endemic. Introducing the raccoon origin virus into the central US could have had serious public health consequences if it had become established and spread within the local raccoon population. The 2024 Omaha CA was estimated to cost approximately $500,000 and consisted of ORV distribution, pet vaccination clinics, trap-vaccinate-release of raccoons, and enhanced surveillance to detect re-appearance of the raccoon RV for up to one-year post-incident [Chipman, personal communication]. This effort prevented potential human rabies exposures and reduced the need for PEP which is the costliest expense in rabies prevention. PEP consists of a series of three anti-rabies vaccines, rabies immunoglobulin injections, and post-bite medical care. These costs vary by location in the US and are not always covered by insurance [24].

### Texas wildlife rabies history

In 1988, a cluster of rabid coyotes infected with the domestic dog rabies virus that was endemic in Mexico was detected in South Texas. This RV, named domestic dog variant in coyotes (DDC-RV), quickly spread into 21 Central Texas counties and by 1995, rabid coyotes were reported just outside the city limits of San Antonio [25]. At the same time, increasing numbers of rabid gray foxes infected with the Texas Fox variant (TF-RV) were detected in West-Central Texas. The fox epizootic at one point encompassed 53 Texas counties including the urban center of Fort Stockton which would prove to add logistical complexity to TF-RV elimination. Emergency state funds released in 1994 created ORV programs implemented in 1995 for coyotes and 1996 for gray fox [26–28]. Federal support and other ground resources were made ready as the Texas programs were being built. Twin Otter airplanes owned by the Province of Ontario were given permission to fly across country to aid in the distribution of the ORV doses in Texas.

From a public health point of view, battling two simultaneous yet geographically distinct rabies epizootics in two different wildlife reservoir species remains an unprecedented chapter in US rabies prevention history. Due to this experience, a true One Health partnership developed between federal, state, and local agencies to effectively combat these epizootics. Of critical importance to the program’s success was development of a laboratory diagnostic test that could differentiate at the molecular level between the coyote and fox RVs [29]. Texas ORV campaigns from 1995 to 2013, plus substantial federal assistance, eliminated these two wildlife RVs and the effort was determined to be cost beneficial. From 1995 to 2006, ORVP cost the state of Texas approximately $68 million USD ($3.8 million USD/yr.) which realized a cost savings of $3.38-$13.12/per state dollar spent [30]. By 2001, the coyote ORV program had cleared the majority of south Texas of the DDC-RV. Based on progress made, the coyote ORV zone changed in size and shape over time to become a barrier along the US-Mexico border.

**Fig 2:**
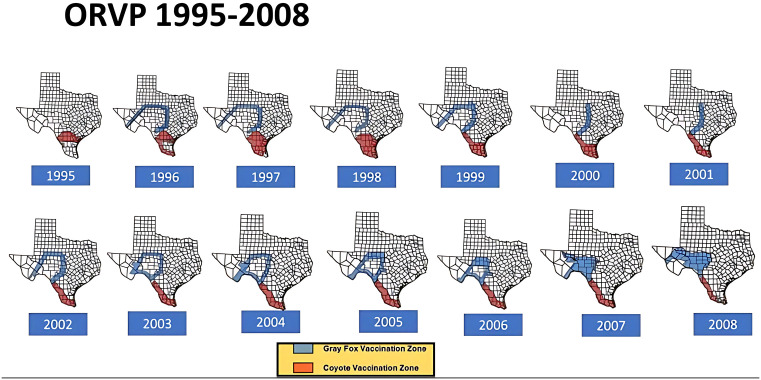
Texas Oral Rabies Vaccination Program Baiting Zones (1995 – 2008). The coyote rabies elimination program began in 1995 in south Texas as a wedge-shaped zone (red areas on map) to prevent northern spread of the canine rabies variant circulating in coyotes (DDC-RV). The DDC-RV zone enlarged in size from 1995-1998 and due to successful reduction in reported cases the zone was reduced in size annually to a become a barrier zone by 2008. The Texas Gray Fox rabies variant (TF-RV) elimination program (blue areas on map) began in 1996 and continued until 2013. The TF-RV zone initially encircled the affected area, except in 2000 and 2001 when reduced funding limited the bait zones to only the eastern barrier. The TF-RV program continued until 2013 (see Fig 3). Map base layer: U.S. Census Bureau TIGER/Line Shapefile (Public Domain). https://www.census.gov/geographies/mapping-files/time-series/geo/cartographic-boundary.html.

**Fig 3:**
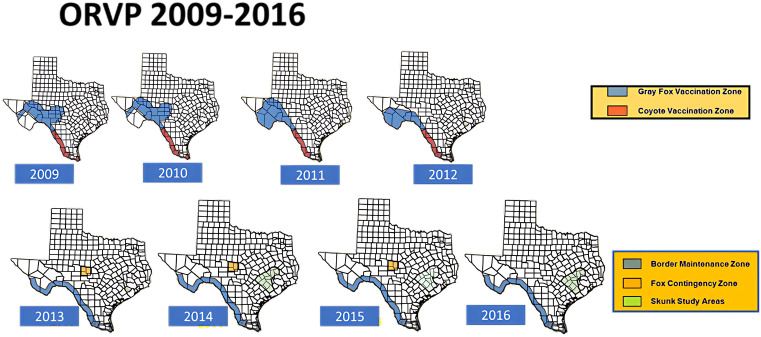
Texas Oral Rabies Vaccination Program Baiting Zones (2009-2015). The bait distribution zones continued to change location over time during the coyote rabies elimination phase (2009-2012). Following elimination of the DDC-RV the coyotes program became the Border Maintenance Zone in 2013. Gray fox bait distribution and contingency actions continued with the last TF-RV reported case in a rabid cow in 2013. This map also includes experimental skunk studies (2014-2016). Map base layer: U.S. Census Bureau TIGER/Line Shapefile (Public Domain). https://www.census.gov/geographies/mapping-files/time-series/geo/cartographic-boundary.html.

**Fig 4:**
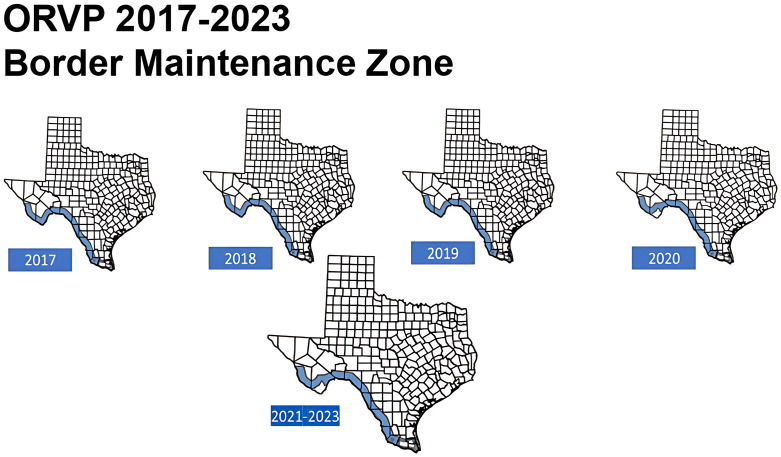
Texas Oral Rabies Vaccine Program bait distribution maps from 2017 through 2023. Following the elimination of the canine rabies variant circulating in coyotes and the Texas gray fox variant, the Texas ORVP continues as a Border Maintenance Zone (BMZ) in southwest Texas along the US/Mexico border (blue areas on map). Map base layer: U.S. Census Bureau TIGER/Line Shapefile (Public Domain). https://www.census.gov/geographies/mapping-files/time-series/geo/cartographic-boundary.html.

Following elimination of the DDC-RV, vaccine doses no longer required in south Texas were shifted northward to intensify ORV efforts on eliminating the TF-RV. Subsequent campaigns in Texas used approximately 2.5 million ORV doses distributed in January of each year (TX-DSHS annual reports 2007–2022) with an estimated annual operational cost of $1.5 M USD. Additional vaccine doses, distribution resources, and indirect support were provided each year by USDA-WS. From 2009 – 2012 the gray fox and coyote programs were managed as separate yet adjoining ORV operations. In 2013, they were combined to create the border maintenance zone (BMZ) which follows the southern and western contours of the Texas border from South Padre to El Paso [28] (Figs 2–4).

In 2013, a rabid cow detected near Brady, TX infected with the TF-RV, resulted in an CA distributing ORV doses and enhanced surveillance in the area surrounding the case. This case was the last report of the TF-RV detected in the US [3]. Since 2014, distribution of approximately 800,000 to 1.2 million ORV doses per year along the west Texas border with Mexico has prevented re-entry of the DDC-RV and kept the emerging Arizona Fox RV (AF-RV) from entering west Texas from New Mexico. As of 2015, no additional CAs have been required along or within the BMZ (Rollo and Parker, personal communication). A small study in east Texas was conducted from 2014-2016 to evaluate bait uptake in skunks. Since 2017, there has essentially not been any substantive changes to the TX-ORVP program.

### Cost effectiveness analysis

The central premise of this study is that the reduction in bait density introduced under the 2023 BMZ framework may increase the probability of barrier breaches. Such breaches could generate higher variable expenditures, including viral surveillance, contingency actions, and the administration of higher levels of PEP, to an extent that negates the fixed-cost savings realized in 2023. Under these circumstances, the 2023 BMZ may no longer remain cost-effective.

Cost-effectiveness analysis provides a systematic approach for evaluating whether the financial benefits of a policy intervention outweigh its associated risks and expenditures. In the context of BMZs, reductions in fixed costs must be assessed against the potential escalation of variable costs arising from increased vulnerability to breaches. Previous studies in health economics and disease control have emphasized that interventions which appear cost-savings in the short term may, under conditions of heightened risk, become economically unsustainable when long-term contingency actions are considered [31–34,35,36].

Accordingly, this study employs CEA to identify the threshold of variable cost increases beyond which the 2023 BMZ framework ceases to be economically viable. By situating the analysis within established principles of cost-effectiveness and risk management, the study aims to provide evidence-based insights into the sustainability of bait density reductions and their implications for both fiscal planning and public health protection.

Previous economic studies have demonstrated a positive public health benefit of ORV application and wildlife rabies prevention programs and used benefit-cost calculations [31]. In this paper, a CEA was used to consider the financial impact of potential rabies incursions on the BMZ. By using fixed and variable economic indicators over time, a CEA can proactively determine the financial impact of public health disease prevention activities including fixed and variable costs.

We conducted a comprehensive overview of rabies control and prevention efforts across various regions and reservoir species, emphasizing the economic evaluations and outcomes of ORV programs. It highlights the use of diverse methodologies, ranging from surveys and interviews to modeling and historical data analysis, to assessments of the cost-effectiveness of rabies interventions. The studies span multiple states in the U.S., including New Jersey [37], Ohio [38], New York [39,40], Texas [30], and California [41], as well as broader national and international contexts [42,43,44].

For raccoons, several studies demonstrate the financial viability of ORV programs. In New Jersey, a benefit-cost (B/C) ratio ranging from 2.2 to 6.8 was reported, with a significant portion of costs attributed to supplemental dog vaccinations [37]. National modeling efforts estimated the cost of establishing and maintaining a rabies barrier at $58–$148 million, with potential benefits reaching up to $496 million [30]. Ohio’s ground baiting strategy proved less expensive, though a formal cost-benefit analysis was not conducted. In New York, consolidated county costs revealed that raccoon control accounted for $0.8 million of a $2.1 million total, with PEP and animal testing (AT) costs varying widely [39].

Texas stands out for its successful coyote-focused ORV program, which yielded B/C ratios between 3.38 and 13.12 over a decade, with estimated benefits ranging from $36 million to $89 million [30]. This underscores the effectiveness of targeted vaccination in high-risk areas. Broader assessments across the U.S. and Canada found that while trap-vaccinate-release (TVR) and point-in-containment (PIC) strategies are costly, ORV remains economically justified with B/C ratios exceeding 1.0 [43]. In California, skunk-focused interventions showed variable cost-effectiveness depending on geographic scope, with expanded coverage in Los Angeles significantly improving the B/C ratio.

More recent studies continue to affirm the value of ORV programs. In New York, a 2016 analysis using net present value (NPV) and regional economic modeling (REMI) found a B/C ratio of 1.71, with benefits largely stemming from avoided PEP and reduced AT costs [39]. A global perspective reinforces the importance of vaccinating reservoir species, noting that elimination of rabies in wildlife is often more cost-effective than relying solely on PEP and diagnostic testing [42]. Overall, while rabies control programs demand substantial resources, their long-term benefits, both economic and public health, are significant. Strategic vaccination of wildlife reservoirs, particularly through ORV, emerges as a cornerstone of effective rabies management across diverse ecological and geographic settings.

Cost effective analyses have been used to examine different strategies in public health with a focus on human interventions [32,33]. In the case of the TX-ORVP, such analyses support decision makers in determining which interventions have contributed the most to improving the health of Texans. Cost-saving is an on-going exercise for most public health programs. The primary drivers for maintaining the TX-ORVP are preventing re-entry of the DDC-RV, reducing spread of new RV variants, and disease surveillance at the US-Mexico border.

We chose to focus on the TX-ORVP BMZ which has been an established entity since 2014. We examined three different scenarios comparing the current BMZ program, and the impact of a translocated rabid animal (i.e., an incursion) into rural and populated areas. There are many limitations of this analysis. First, fixed costs are likely to change over time. Baits, fuel, and administrative costs will at the very least increase by inflation. Fuel cost changes are uncertain but are very likely to increase in the future and these changes can be substantial. Second, we have omitted cost impacts related to rabies laboratory testing, livestock, or companion animals in this analysis. In the event of a breach in the barrier, livestock could be impacted by wildlife rabies as well as companion animals and laboratory costs would increase. Lastly, the fear of the entry of the DDC-RV rabies could have psychological impacts on people in the affected region that may be borne out in ways that impact their economic health. Therefore, we believe the estimate of economic impacts of wildlife rabies translocations described in this paper are conservative.

## Methods

Data from Texas Department of State Health Services (TXDSHS), Austin, Texas records consisted of ORVP operational costs, rabies cases by species, and cost of implementing rabies outbreak control [i.e., CAs, and estimates of PEP]. This analysis did not include indirect support provided by other agencies, nor did it include any in-kind support from any source. The reason for limiting this analysis to state-derived funds is to demonstrate the costs and benefits at the state level. In Texas, PEP is offered to people following a potential rabies exposure by TXDSHS as well as private doctors. Medical care for bite victims is performed by numerous primary care physicians, hospitals, and urgent care centers. Estimates of PEP used in this analysis represent only a fraction of total administrations per year in Texas due to the privatization of such data. During 2021, PEP was administered by the state to 261 people, of whom 50 (19.2%) acquired the biologicals from DSHS PHR offices and 211 (80.8%) from local health department depots. The reported cost of the biologicals was $976,463 distributed to 167 people (64.0% of people receiving biologicals from TXDSHS inventory) at a cost of $839,137 and an average cost of $5,025 per person (median: $4,899; range: $2,087-$8,502). Rabies biologicals were distributed to 258 (98.9%) Texas residents and 3 (1.1%) out of state residents [34].

Texas Consumer Price Index (CPI) data were publicly sourced from the Social Security Administration. All costs marked as “adjusted” are based on the 2023 annual average CPI, as that is the most recent and complete year of data at the time of analysis. The computing software used was RStudio, version 4.1.2 (2021-11-01).

Annual state ORVP total costs (TC_i_) for scenario i consisted of the fixed costs (C_F_) and variable costs (C_V_). To calculate C_F_, we collected data for baits quantities distributed per year, number of wildlife specimens tested per year, annual ORVP operational cost, and lab fees for rabies testing. To calculate C_V_, we used values averaged from 2019-2021 for rabies surveillance testing, human PEP administrations, and the average cost of a CA. The Texas ORVP experienced three CA during the DDC-RV elimination phase of the program (1995 until 2007) (see below). The TF-RV elimination phase was from 1996-2013. The establishment of the BMZ was considered to start in 2014 and has continued without a breach requiring a CA until the present day (2025).

Due to the financial impact emergency responses have on disease prevention costs, details of past TX-ORVP CAs were considered. The first CA (Rankin County, 2007) cost $38,943. The cost was $10,859 for baits, $1,100 for fuel, $14,235 for surveillance, $11,250 travel and other costs at $1,500. The second CA (Pecos River, 2007) cost $671,843. This cost was $424,995 baits, $13,900 for fuel, $34,451 for travel, $150,797 for program surveillance, Balmorhea area enhanced surveillance ($13,650), Pecos enhanced surveillance ($24,050), and other costs ($10,000). The third CA (Concho/McCulloch Cow 2013) cost $414,200. This cost was $343,100 for baits, $8,000 for fuel, $58,100 for total surveillance, and other costs ($5,000). The associated cost breakdown shows 84% of cost allocated to baits, 11% allocated to surveillance distribution, 2.3% to fuel, 2% to travel, and 0.7% to other expenses. The 2007 Rankin and Pecos River CAs resulted in 51 people receiving PEP costing $42,099, while the 2013 Concho/McCulloch Cow CA resulted in 15 people receiving PEP costing $38,079. We used the average ($375,000) of these three events as a cost estimate of CAs for this analysis.

To calculate total costs (TC) of the program for all scenarios,


$$TC=C_{F}+C_{V}$$ (1)

C_F_ is the summation of oral vaccine baits (OVB), distribution (DIST), fuel (F), and administrative (A) costs.


$$C_{F}=OVB\;+\;DIST\;+\;F\;+\;A$$ (2)

For this analysis, we assumed that fixed costs (C_F_) were close to or at the minimum allowable amount to retain use of the resource, so we did not vary these costs for this analysis. This analysis focuses only on examining the impacts of potentially changing variable costs.

C_V_ is the summation of viral surveillance (VS), post-exposure prophylaxis (PEP), and contingency actions (CA).


$$C_{V}=VS\;+\;PEP\;+\;CA$$ (3)

Total cost for the baseline was compared to the TC for each scenario (i). TC_Baseline_ is derived from annual C_F_ and C_V_ averaged from 2019-2021. Prior to the BMZ modifications of 2023 the average total Baseline ORVP C_F_ (2019–2021) were just over $1.85 million and average C_V_ were almost $600k for a total average annual cost of just over $2.4 million. In 2023, the BMZ was modified, with C_F_ costs falling from fewer baits utilized (C_F-Baseline_>C_F-2023_) and resulted total annual costs were just under $2 million. To determine the cost effectiveness, three hypothetical scenarios were analyzed.

The first scenario (i = 1) considers the current state of ORV BMZ based on C_F2023_. Following the elimination of the DDC-RV in 2007 and TF-RV in 2013 and establishment of the ORV BMZ in 2014, no terrestrial wildlife rabies cases have entered the US across the US Mexico border since the implementation of the program and annual bait distributions maintain the barrier’s integrity. Rabies incursion risks to the barrier still exist due to the lack of wildlife rabies surveillance in Mexico and emerging fox rabies cases in New Mexico. Therefore, it is logical to want to increase wildlife rabies surveillance on the Texas side of the BMZ. VS represents surveillance for the DDC-RV at the Texas–Mexico border and is routine, proactive, and long-term and designed to prevent reintroduction of this strain. In contrast, surveillance during a CA is reactive, targeted, and intensified, triggered by evidence of a rabies outbreak or spillover event. Therefore, the costs associated with VS are not incorporated into CA. Scenario 1 recognizes this situation and models varying levels of viral surveillance a component of C_V_, (Equation 4).


$$C_{V\text{1}}=\alpha VS+\overset{―}{PEP}$$ (4)

Where α is a scalar on viral surveillance, PEP is held constant at the 2023 level and there are no CAs. The resulting TC_1_ = C_F2023_ + C_V1_ can then be compared to TC_Baseline_ to determine economic efficiency.

The second and third scenarios consider the impact of state ORV budget reductions. In 2000 and 2001 state ORV funds were significantly reduced. To compensate for this budget shortfall, dollars used in the coyote program were stretched to cover both the coyote and gray fox ORV zones. Reducing the width of the barrier or the density of baits distributed in the BMZ potentially increases the probability of a breach of the barrier and the need for a CA to eliminate the impacts of the breach. Average CA costs of $375k were utilized in Scenario 2, to model the potential impact on economic efficiency of actions related to a breach in the BMZ. Since a CA represents a breach in the barrier, this scenario includes a 25% increase in viral surveillance for each emergency response.


$$C_{V\text{2}}=\alpha VS+\overset{―}{PEP}+\gamma CA$$ (5)

Where α is a scalar on viral surveillance and γ is a scalar on contingency actions. When γ = 0 then α = 0, and when γ = 1 then α = 25%, so each increase in gamma results in a 25% increase in alpha.

The BMZ prior to 2023 had been successful at preventing the reemergence of DDC variant rabies, suppressing the number of PEP cases, and necessitating a low level of viral surveillance.

Scenario 3 considers the need for a CA, increased surveillance, and increased PEP cost. This last scenario recognizes that changing the BMZ may heighten the need for all three variable costs due to a breach in the zone. A breach in the barrier increases the threat to human health, typically leading to in increased levels of PEP in the area. Scenario 3 models this relationship between CAs, increased viral surveillance, and increased the threat to humans by increasing all C_V_ by α.


$$C_{V\text{3}}=\alpha (VS+PEP+CA)$$ (6)

Cost effective scenarios are all considered in 2023 dollars and represented by any scenarios that result in a TC_i_ < TC_Baseline_.

## Results

Examining the results by scenario provides interesting insight into the cost effectiveness of the Texas ORVP BMZ. Scenario 1 examined the situation in which the level of wildlife rabies surveillance (i.e., testing and reporting of rabid animals by diagnostic laboratories) one aspect of variables costs, was increased to enhance detection of any incursions or translocations through the BMZ_2023_ while PEP costs remain the same and no CAs were needed. This scenario models the likelihood that to monitor the effectiveness of the 2023 BMZ, which had been reduced in size from previous years, a higher level of surveillance would be necessary to validate the effectiveness of the reduced zone. Results indicate that viral surveillance costs could increase 155% before those costs render the BMZ_2023_ cost inefficient. In other words, the reduced BMZ_2023_ is no longer cost effective if increased surveillance costs exceed 155% causing TC_1_ > TC_Baseline_.

Scenario 2 models the need for an emergency due to a breach or translocation event into a rural area without significant human or domestic animal rabies expenses. If a CA was needed, this analysis assumed that this event would necessitate a 25% increase in viral surveillance. Under this scenario, more than one contingency action results in TC_2_ > TC_Baseline_ and the BMZ_2023_ no longer being cost effective.

Scenario 3 represents the most comprehensive and likely realistic examination of the BMZ_2023_. This scenario captures the interrelatedness of these three variables. If a CA is warranted, then an increase in rabies case surveillance will likely be initiated which means there is likely an increased threat to humans from the breach in the BMZ which would potentially increase PEP expenditures. The results for this scenario indicate that because of the high costs associated with CAs and increases in surveillance and PEP, the BMZ_2023_ is not cost efficient (TC_3_ > TC_Baseline_), and it would be economically more efficient to continue ORVP practices and level of expenditures for the BMZ prior to 2023.

It was assumed for this analysis that fixed costs remained static to reflect that these costs are at or near their minimum to be utilized by the program. Variable costs consisted of viral surveillance costs, PEP costs and CA costs. Our results indicate that there are situations in which the BMZ_2023_ remains cost effective, however, any scenario requiring more than one CA would render the BMZ_2023_ cost inefficient.

## Discussion

Determining the long-term economic value of the Texas ORVP BMZ was the goal of this analysis. The TX-ORVP program, as part of the larger USDA-NRMP, has been quite successful. Eliminating two wildlife RVs required 18 years but the program controlled the outbreaks and continues to mitigate costs associated with wildlife rabies. Since 2017, there has been almost no changes in the Texas ORVP practices or BMZ location and concurrently, no confirmed cases of DDC-RV or TF-RV rabies in the US. Consequently, no animals have tested positive for either variant of rabies on the Texas side of the barrier. By almost any measure, the BMZ has successfully and consistently prevented the reemergence of DDC rabies in the US from Mexico, preventing millions of dollars of impacts to the state of Texas [42].

Today, the public health benefit of preventing the re-entry of wildlife RVs into the US may not be attributed, necessarily, to the ongoing success of the BMZ barrier. This attribution bias can lead to complacency by the general public and an undervaluation of this program by policymakers. The potential for state and federal budget cuts to zoonotic disease prevention efforts are today top of mind. This CEA proactively provides insight when considering potential cost-savings measures (e.g., reduce the number of baits utilized each year, reduce bait density in certain areas of the BMZ, or possibly abandoning baiting altogether in select areas of the BMZ). Additionally, the societal benefits of not having two additional wildlife RVs in Texas cannot easily be assigned a numerical value. Residents outside of Texas also enjoy these benefits since coyotes and gray foxes are common terrestrial species found across the continental US. Expansion of either of these epizootics beyond state lines could have had dire regional public health consequences.

## Conclusion

Preventing infectious diseases is cost effective and cost efficient when compared to suppressing an epidemic [45]. In Texas, the BMZ has previously been shown to be cost efficient, and its benefits exceed the costs of maintaining the zone [30]. The Texas ORVP is a successful, long-term public healthcare investment in disease control and rabies response preparedness. Regular reporting of the economic value of long-standing disease prevention programs increases their visibility with stakeholders and decision makers. The TX ORVP has proven to be an on-going economic public health value to the state of Texas as well as the United States. This analysis demonstrates that while reductions in bait density within the TX‑ORVP barrier maintenance zone can generate short‑term cost savings, these savings are highly sensitive to increases in variable costs and the occurrence of rabies incursions. Enhanced surveillance alone remains economically favorable across a wide range of cost increases, but any scenario requiring contingency actions rapidly eliminates the financial advantages of the 2023 bait‑density reduction. These findings highlight the narrow margin within which reduced‑intensity ORV strategies remain cost‑effective and emphasize the substantial economic risks associated with diminished protection against wildlife rabies spread. As wildlife reservoirs continue to pose persistent zoonotic threats in the United States, sustained and adequately resourced ORV programs remain essential for maintaining rabies‑free zones, preventing costly outbreaks, and safeguarding public health.
